# Supplementary material for: The causal role of circulating inflammatory markers in osteoporosis: a bidirectional Mendelian randomized study
Source: Front Immunol. 2024 Jul 18;15:1412298. doi: 10.3389/fimmu.2024.1412298 (PMC11291241; doi:10.3389/fimmu.2024.1412298)
Supplement: Supplementary file 1 [file DataSheet_1.zip › supplementary material/CIM-OSTEOPOROSIS /GCST90274845.csv_forest.pdf]

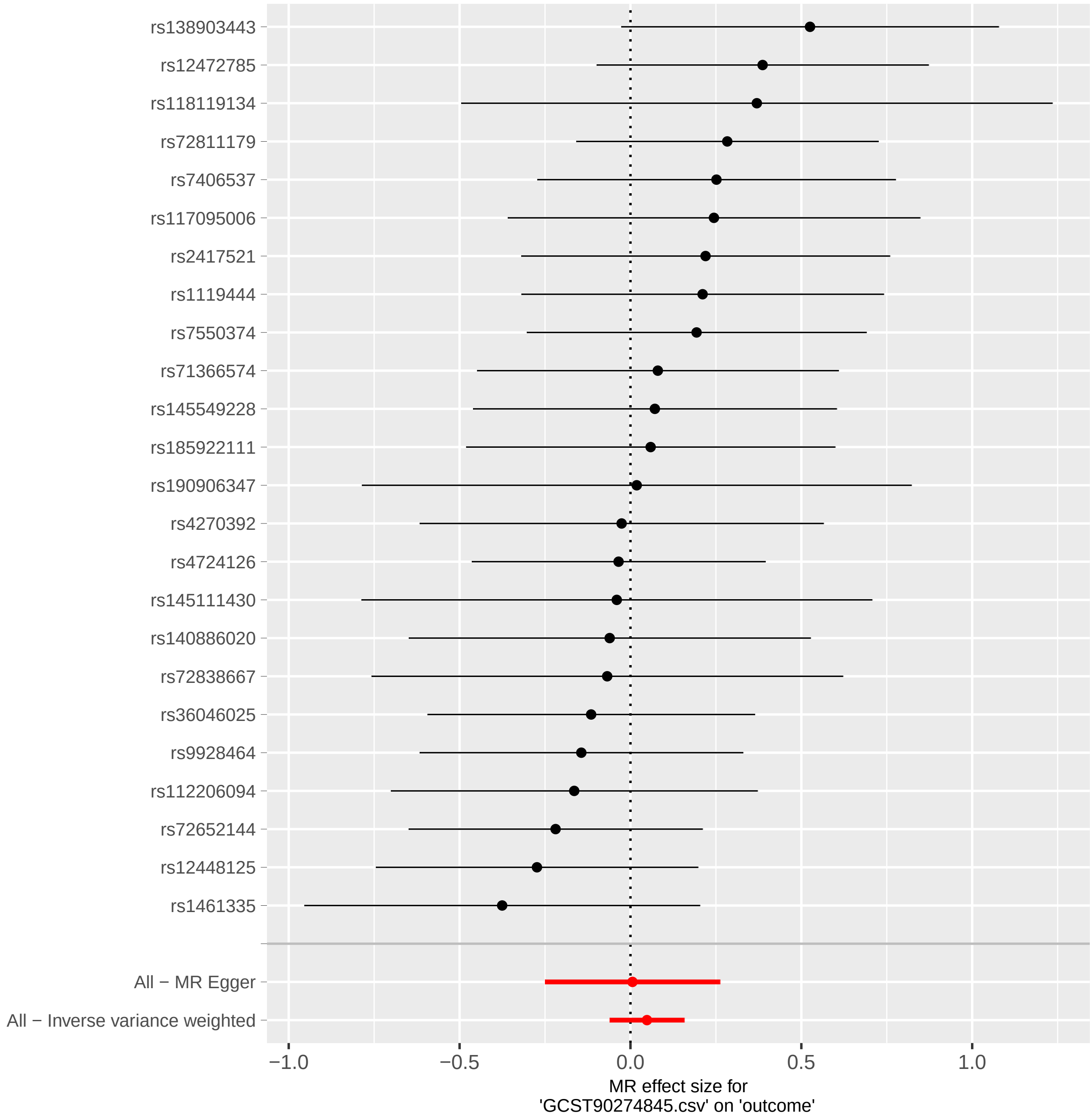
rs138903443
rs12472785
rs118119134
rs72811179
rs7406537
rs117095006
rs2417521
rs1119444
rs7550374
rs71366574
rs145549228
rs185922111
rs190906347
rs4270392
rs4724126
rs145111430
rs140886020
rs72838667
rs36046025
rs9928464
rs112206094
rs72652144
rs12448125
rs1461335
All − MR Egger
All − Inverse variance weighted
−1.0
−0.5
0.0
0.5
1.0
MR effect size for
'GCST90274845.csv' on 'outcome'
